# Protection against Ischemia-Induced Oxidative Stress Conferred by Vagal Stimulation in the Rat Heart: Involvement of the AMPK-PKC Pathway

**DOI:** 10.3390/ijms131114311

**Published:** 2012-11-05

**Authors:** Shan-Shan Kong, Jin-Jun Liu, Xiao-Jiang Yu, Yi Lu, Wei-Jin Zang

**Affiliations:** Department of Pharmacology, College of Medicine, Xi’an Jiaotong University, Xi’an 710061, China; E-Mails: kongshanshan@stu.xjtu.edu.cn (S.-S.K.); jupet@163.com (J.-J.L.); yxs@mail.xjtu.edu.cn (X.-J.Y.); lyppon@stu.xjtu.edu.cn (Y.L.)

**Keywords:** acute myocardial infarction, vagal stimulation, reactive oxygen species, oxidase stress, muscarinic acetylcholine receptor

## Abstract

Reactive oxygen species (ROS) production is an important mechanism in myocardial ischemia and nicotinamide adenine dinucleotide phosphate (NADPH) oxidase is one of major sources of ROS in the heart. Previous studies showed that vagus nerve stimulation (VNS) is beneficial in treating ischemic heart diseases. However, the effect of VNS on ROS production remains elusive. In this study, we investigated the role of VNS onischemia-induced ROS production. Our results demonstrated that VNS alleviated the myocardial injury, attenuated the cardiac dysfunction, reserved the antioxidant enzyme activity and inhibited the formation of ROS as evidenced by the decreased NADPH oxidase (Nox) activity and superoxide fluorescence intensity as well as the expression of p67phox, Rac1 and nitrotyrosine. Furthermore, VNS resulted in the phosphorylation and activation of adenosine monophosphate activated protein kinase (AMPK), which in turn led to an inactivation of Nox by protein kinase C (PKC); however, the phenomena were repressed by the administration of a muscarinic antagonist atropine. Taken together, these data indicate that VNS decreases ROS via AMPK-PKC-Nox pathway; this may have potential importance for the treatment of ischemic heart diseases.

## 1. Introduction

Acute myocardial infarction (AMI) is a major cause of morbidity and mortality worldwide, which is associated with increasing production of reactive oxygen species (ROS) [1] such as superoxide. Vanden *et al.* have reported that cultured cardiomyocytes generate significant ROS during ischemia and ROS generation contributes significantly to the cellular injury seen at reperfusion [2]. Furthermore, ROS induce a variety of cardiomyocyte abnormalities including cell death and apoptosis [3].

ROS may emanate from several sources, including nicotinamide adenine dinucleotide phosphate (NADPH) oxidase, xanthine oxidase, nitric oxide synthase and mitochondrial cytochromes. Although each of these can contribute to the oxidative burden, evidence is accumulating that the predominant superoxide-producing enzyme in heart is NADPH oxidase (Nox), which is important in signal transduction-dependent ROS [4,5]. The Nox system comprises seven members, including Nox1-5, Duox1 and Duox2. However, the main Nox isoforms expressed in cardiac tissue are Nox2 [6], which are implicated in ischemia [7,8] and heart failure [9], as well as cardiac hypertrophy [10]. Numerous studies have indicated that Nox-derived ROS plays an important role in the pathophysiology of many cardiovascular diseases. Thus, inhibition of Nox-derived ROS production might be a useful strategy for treating myocardial ischemia diseases.

It is recognized that an imbalance of the autonomic nervous system is involved in myocardial ischemia [11]. Increased sympathetic activity and reduced vagal activity contribute to increased mortality both in AMI and heart failure [12,13]. Recently, studies from several labs including our own have reported that vagus nerve stimulation (VNS) inhibits inflammation factors release [14], modulates myocardial remodeling and markedly improves survival after myocardial infarction in rats [15], as well as improving cardiac function in heart failure patients [16], suggesting an ameliorative effect of direct neural interventions against ischemic heart diseases. With regard to life-threatening arrhythmias in rats with acute ischemia, VNS has been reported to prevent ventricular fibrillation in rats by the prevention of the loss of phosphorylated Cx43 [17]. In addition to this, NO may be involved in the antifibrillatory effect of VNS [18]. This study has been undertaken in order to evaluate the role of VNS treatment on cardiac oxidative stress in rats with AMI. Furthermore, we evaluated the possible underlying signaling mechanism with a special focus on AMPK-PKC signaling, which is involved in the Nox-mediated ROS production [19,20].

## 2. Results and Discussion

### 2.1. Vagal Stimulation Attenuated Myocardial Injury and Improved Cardiac Function

**Figure 1:**
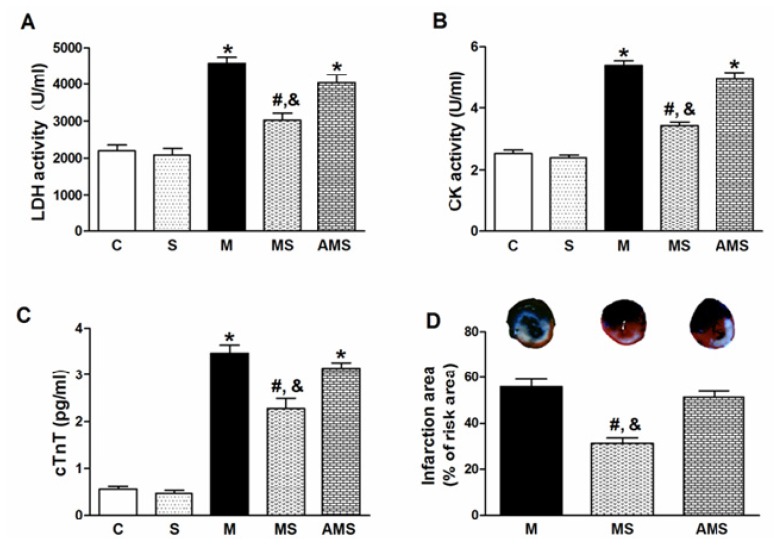
Vagal stimulation decreased susceptibility of myocardial injury. The animals were randomized into five groups: C indicates sham-operated rats with sham stimulation; S depicts rats treated with only right vagus nerve stimulation (VNS); M indicates acute myocardial infarction (AMI) rats treated without VNS; MS represents AMI rats treated with VNS while AMS means the acute myocardial infarction rats with both vagal stimulation and atropine sulfate. (**A**) Serum levels of LDH from each group; (**B**) Serum levels of creatine kinase (CK) from each group (*n* = 12 for each group); (**C**) Serum levels of cTnT from each group; (**D**) Reduction in AMI-induced myocardial infarct size (percentage risk area) by VNS. Note: the blue color is Evans blue dye; the red color is 2,3,5-triphenyltetrazolium chloride staining corresponding to the uninfarcted area. The white color corresponds to the infarcted area. Data are the mean ± SEM (*n* = 6). *p < 0.05 compared with control (C); **#**, & *p* < 0.05 compared with M and AMS, respectively.

In the present study, we stimulated the right vagus nerve following the previous experience [14,15] and employed a new stimulation parameter that lowering the heart rate (HR) by 10% compared to baseline level. Figure 1A–C shows serum levels of lactic dehydrogenase (LDH), creatine kinase (CK) and cardiac troponin T (cTnT) in all groups. The model rats (M group) exhibited a significant serum enzyme rise compared with the control (C) group. In the MS group, subjected to VNS during AMI, serum LDH, CK as well as cTnT activity were significantly decreased compared with the M group. However, the cardioprotective role of VNS was partially offset by atropine (atro) in the AMS group, subjected to atropine and VNS during AMI. Figure 1D shows representative sections of hearts after AMI in M, MS and AMS groups, respectively. Quantitative comparison of infarct size in the M, MS and AMS groups demonstrated a significant difference in infarct size among the three groups, similar to the results of serum enzymes level.

**Figure 2:**
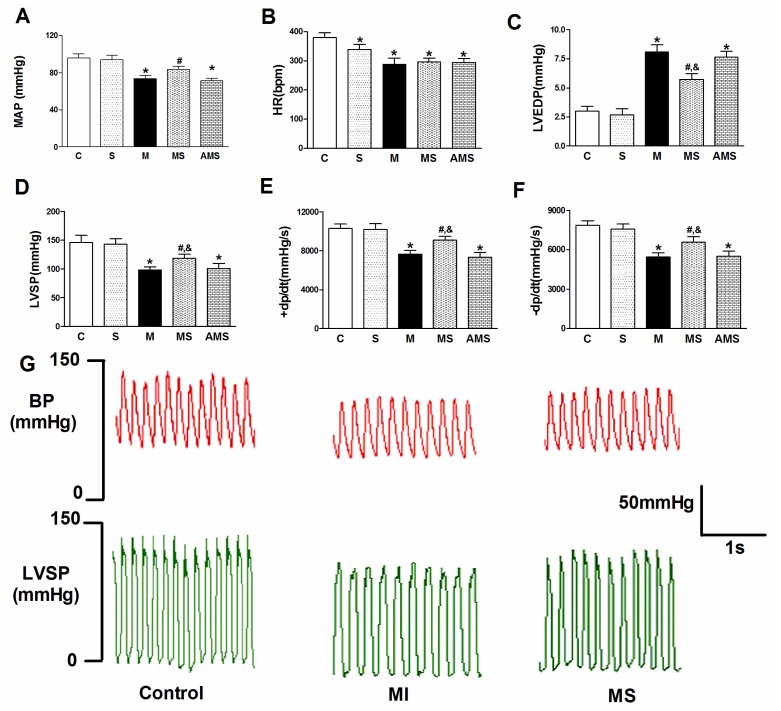
Effect of vagal nerve stimulation (VNS) on cardiac function. (**A**) MAP, mean arterial pressure; (**B**) HR, heart rate; (**C**) LVSP, left ventricular systolic pressure; (**D**) LVEDP, left ventricular end-diastolic pressure; (**E**) +d*P*/d*t* = maximal slope of systolic pressure increment; (**F**) −d*P*/d*t* = maximal slope of diastolic pressure decrement; (**G**) Raw pressure data collected in C, M and MS group at the end of experiment. Data are the mean ± SEM (*n* = 12). *p < 0.05 compared with control (C); **#**, & *p* < 0.05 compared with M and AMS, respectively.

As shown in Figure 2, there were no significant differences between the C group and S (vagal stimulation alone without AMI) group with respect to the heamodynamic measurements. Cardiac function was better and stable as indicated by the mean arterial pressure (MAP), left ventricular systolic pressure (LVSP), contractility (maximal slope of systolic pressure increment, +d*p*/d*t*_max_) and diastolic pressure (left ventricular end-diastolic pressure, LVEDP), while the administration of atropine resulted in a decreases of cardiac function. These results indicated that direct parasympathetic activation by VNS could induce positive effects on myocardial injury and left ventricular function in the present study.

### 2.2. Changes of Serum Oxidant and Antioxidant Enzyme Activities in Each Group

**Figure 3:**
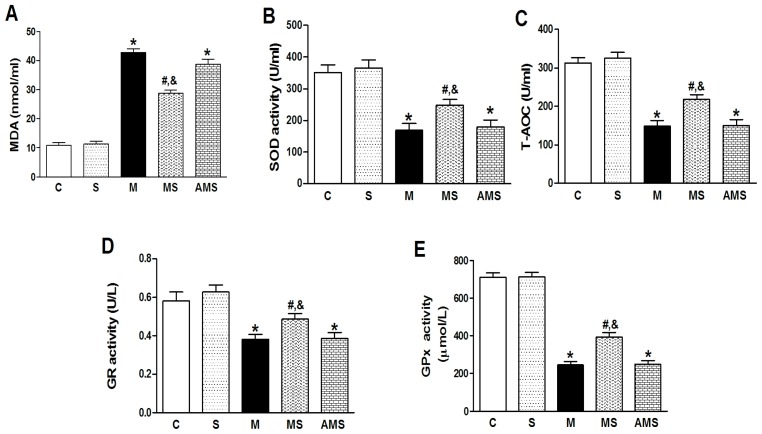
Changes of serum MDA, SOD, T-AOC, GR and GPx levels in each group. (**A**) MDA; (**B**) SOD; (**C**) T-AOC; (**D**) GR; and (**E**) GPx levels in serum, respectively. Data shown are mean ± SEM (*n* = 6). *p < 0.05 compared with control (C); **#**,& *p* < 0.05 compared with M and AMS, respectively.

Increased contents of serum malonaldehyde (MDA) and decreased levels of serum superoxide dismutase (SOD), total antioxidant capability (T-AOC) and glutathione reductase (GR) as well as glutathione peroxidase (GPx) had been induced in AMI rats compared to C groups (Figure 3). However, VNS reduced MDA contents and increased SOD, T-AOC, GR as well as GPx levels. After administration of atropine, the beneficial role of VNS was depleted. These results showed that VNS can reverse the imbalance of oxidatant-antioxidant system induced by myocardial ischemia.

### 2.3. Vagal Stimulation Inhibited Oxidative Stress in Rats with AMI Mainly via the NADPH Pathway

**Figure 4:**
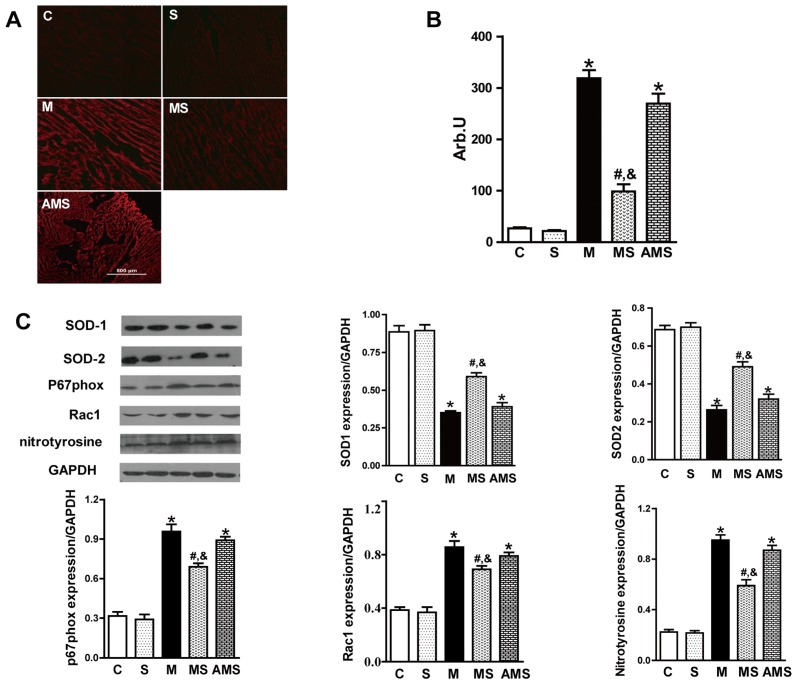
Effect of vagal stimulation on oxidative stress in rats with AMI. Rats in each group were sacrificed at the end of experiment and serum was collected. (**A**) Representative of DHE fluorescence staining for superoxide in LV tissue sections; (**B**) Fluorescent intensity of superoxide in each group; (**C**) Representative of SOD1,SOD2, p67phox, Rac1 and nitrotyrosine expression and their respective quantification in control; Data shown are mean ± SEM (*n* = 5). *p < 0.05 compared with control (C); **#**,& *p* < 0.05 compared with M and AMS, respectively.

We first determined the intracellular ROS content in frozen ventricular tissue sections from rats in each group. Dihydroethidium (DHE) fluorescence staining, a relatively specific marker of superoxide, is significantly stronger in AMI group compared to controls (Figure 4A). As expected, the intensity of fluorescent signal in the MS group was significantly reduced relative to the M groups (Figure 4B).

It has been reported that SOD serves as a major antioxidant enzyme responsible for the first step of the superoxide removal system [21]. SOD consists of three enzymatic isoforms: cytosolic CuZn-SOD (SOD1), mitochondrial Mn-SOD (SOD2) and extracellular CuZn-SOD (SOD3). However, SOD3 is expressed only in a limited number of tissues (lung, kidney, and fat tissue) [22]. Thus, in the present study, we only focused on the effect of VNS on the SOD1 and SOD2 protein expression. Obviously, VNS enhanced the expression of SOD1 and SOD2 in rat myocardium (Figure 4C).

Since it has been reported that the predominant superoxide-producing enzyme in heart is Nox, we analyzed the expression of Nox associated subunits, p67phox and Rac1. We also analyzed the expression of nitrotyrosine, which is also an important molecular indicator of ROS. VNS evidently inhibited the expression of p67phox, Rac1 as well as nitrotyrosine (Figure 4C). However, atropine significantly facilitated the upregulation of p67phox, Rac1 and nitrotyrosine expression.

**Figure 5:**
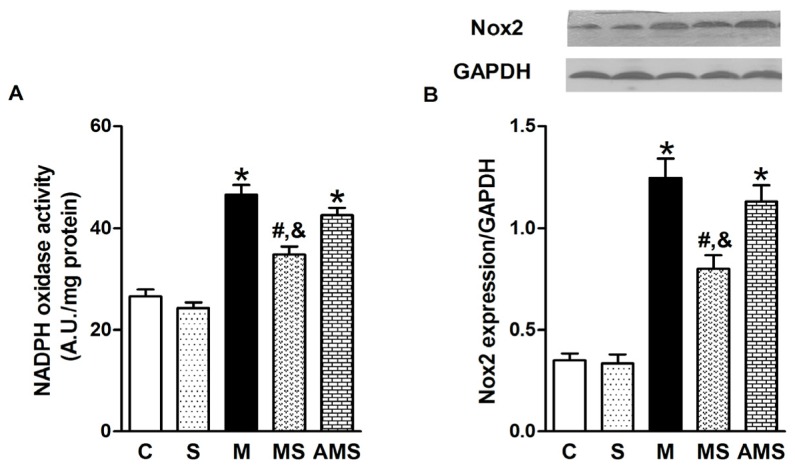
VNS inhibits oxidative stress in rats with AMI via Nox pathway. (**A**) Nox activity measured by lucigenin-enhanced chemiluminescence in each group; (**B**) Representative of Nox2 protein and analysis in each group. Data shown are mean ± SEM (*n* = 5). *p < 0.05 compared with control (C); **#**,& *p* < 0.05 compared with M and AMS, respectively.

Then we determined whether the increase in ROS production is attributable to the activation of Nox, a superoxide-producing enzyme. After myocardial infarction, Nox activity was significantly increased compared to control group; however, VNS markedly decreased the Nox activity while atropine, an antagonist of muscarinic receptor, increased the activity of Nox activity (Figure 5A).We also measured the protein expression of Nox2, which is the key Nox isoforms in the cardiomyocytes (Figure 5B). The results demonstrated that Nox was involved in myocardial ischemia-induced ROS production, implying that vagal stimulation inhibited oxidative stress in rats with AMI via the Nox pathway. Roe *et al.* have demonstrated that inhibition of Nox alleviated experimental diabetes-induced myocardial contractile dysfunction [23]. Our findings on myocardial dysfunction related to AMI can be explained, at least in part, by the formation of ROS which is thought to exert negative inotropic effects [24].

### 2.4 Effects of Vagal Stimulation on AMPK-PKC Pathway

**Figure 6:**
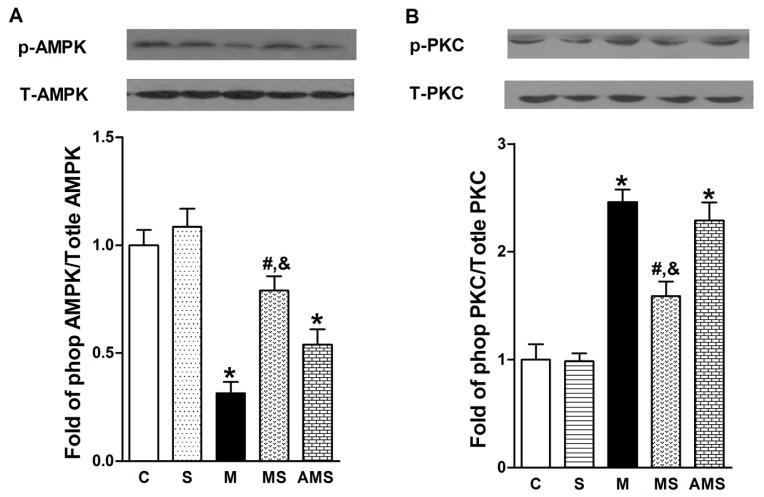
Vagal nerve stimulation modulated ROS generation via AMPK-PKC expression. (**A**) Representative of Western blots for phosphorylated and total AMPK expression from each group; (**B**) Representative of Western blots for phosphorylated and total PKC expression from each group. Data shown are mean ± SEM (*n* = 5).*p < 0.05 compared with control (C); **#**,& *p* < 0.05 compared with M and AMS, respectively.

AMPK can reverse and alter many cellular pathways to protective against the oxidative injury [25]. We assume that myocardial ischemia-induced ROS generation was caused by the regression of AMPK phosphorylation. To verify our hypothesis, the protein expression of AMPK phosphorylation was evaluated by western blot analysis. As shown in Figure 6A, AMPK phosphorylation was decreased in AMI group compared with control group. In contrast, VNS significantly increased the phosphorylation of AMPK. However, atropine antagonized the protective role conferred by VNS.

Furthermore, previous studies have shown that PKC isoforms play a key role in the regulation of Nox subunit [26] and AMPK-α can inhibit the ROS production through the suppression of PKC, which in turn prevents activation of Nox [27]. Therefore, we focused on our attention on the effect of VNS on PKC phosphorylation. As shown in Figure 6B, myocardial ischemia facilitated the PKC phosphorylation compared with control group while VNS significantly decreased PKC phosphorylation level. However, atropine prevented the VNS-induced reduction of phosphorylation PKC. These results demonstrated that AMPK-PKC pathway might be the molecular basis of the VNS-mediated cardioprotective role.

## 3. Experimental Section

### 3.1. Animals

Adult male Sprague-Dawley rats (220–250 g) were purchased from the Experimental Animal Centre of Xi’an Jiaotong University. All experimental procedures were in accordance with the Guidelines on the Care and Use of Laboratory Animals [28] and were approved by the Ethics Committee of Xi’an Jiaotong University.

### 3.2. Heamodynamics Measurements and Induction of Acute Myocardial Infarction

Rats were anaesthetized with urethane intraperitoneally (1–1.2 g/kg). Rats were then tracheotomized, intubated and ventilated mechanically. Arterial pH, PO_2_ and PCO_2_ were maintained within the physiological range by supplying oxygen and changing the respiratory rate. A polyethylene catheter (PE-50, Becton Dickinson), containing saline solution with heparin, was placed in the left ventricle via the right carotid artery and connected to a pressure transducer to monitor left ventricular (LV) systolic pressure (LVSP), LV end-diastolic pressure (LVEDP) and the maximum rate of increase/decrease of LV pressure (±d*p*/d*t*_max_). Another heparin-filled PE-50 was inserted into the right femoral artery to record arterial pressure. An instantaneous heart rate (HR) was measured from the lead II electrocardiogram (ECG). Experimental solutions were infused started with coronary artery ligation through another PE-50 tubing, which was cannulated into the right femoral vein. Atropine sulfate (1 mg/kg) was administered as a muscarinic receptor antagonist [17].

AMI was established by ligation of the left anterior descending artery (LAD), as described previously [29]. AMI was deemed successful on the basis of regional cyanosis of the myocardial surface distal to the suture, accompanied by the elevation of the ST segment on the electrocardiogram [30].

### 3.3. Vagus Nerve Stimulation

**Figure 7:**
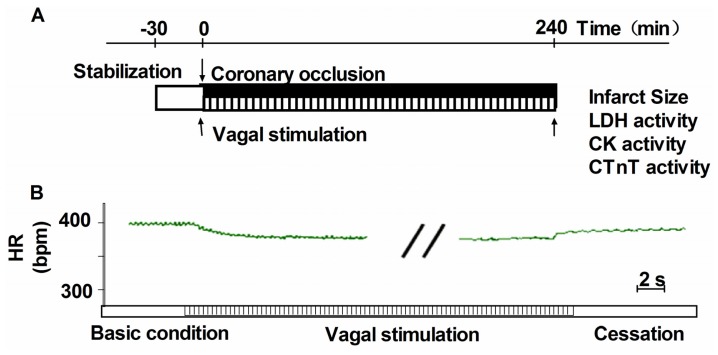
Schematic representation of the protocol and changes in HR with VNS. (**A**) Experimental protocol. After initial surgical preparation, rats were allowed 30 min stabilization. The AMI model was established by ligation of the left anterior descending artery (LAD), VNS was started with coronary artery ligation and continued for the 240 min of artery ligation; (**B**) Changes in HR with VNS. During the VNS, HR was reduced by 10% from baseline, indicating that the effective of VNS; while after the cessation of VNS, HR almost returned to the baseline level, indicating the effective role of VNS.

Bilateral cervical vagus nerves were identified and transected at the neck region. A pair of bipolar electrodes was attached at the cardiac end of the right vagus nerve. Only the distal end of the vagal nerve was placed on a pair of platinum wires and was used for stimulation [17]. The electrode was connected to an isolated constant voltage stimulator (ML866, Power Lab, AD Instruments, Australia). The vagus nerve was stimulated with electrical rectangular pulses of 1ms duration at 2 Hz during LAD ligation. The electrical voltage of pulses was optimized in each rat to obtain a 10% reduction in HR started with LAD ligation. The actual electrical voltage was in the range of 2 to 4 V. VNS was performed with LAD ligation and continued for 240 min after LAD ligation (Figure 7A). To prevent drying and to provide insulation, the stimulation electrodes and the vagus nerve were immersed in a mixture of white petrolatum (vaseline) and paraffin [31,32].

To ensure the effectiveness of our VNS, we monitored HR during the experimental protocol. As shown in Figure 7B, a 10% reduction in HR was seen with VNS and this effect of VNS was completely reversible because HR returned to the pre-stimulation levels within 20 s after cessation of VNS.

### 3.4. Exclusion Criteria

Rats were excluded from the study if: (1) intractable severe arrhythmia occurred; (2) the rat died during the surgical procedures and responded poorly to VNS; and (3) a sustained fall in MAP to <60 mmHg was observed [14].

### 3.5. Blood Sampling and Tissue Preparation

Each animal had 2 mL of whole blood rapidly withdrawn via the arterial catheter into a syringe. Blood samples were taken before 5 min at the end of experiment.

At the end of the experiment, the animals were euthanized. The whole heart was quickly excised and washed with cold phosphate buffer consisting of (in mM): 137 NaCl, 2.7 KCl, 10 Na_2_HPO_4_, and 2 KH_2_PO_4_ at pH 7.4. Then right ventricular free wall, and atrial appendages were dissected away, the remaining left ventricular wall was snap frozen in liquid nitrogen and stored at −80 °C.

### 3.6. Cardiac Specific Injury Markers Measurement

Blood was collected into serum separator tubes (Becton Dickinson, Rutherford, NJ, USA) and serum was obtained by centrifugation at 6000*g* for 6 min. Serum levels of LDH and CK were evaluated according to the manufacturer’s instructions (Nanjing Jiancheng Biological Technology, Nanjing, China). Serum level of cTnT was measured by a commercial kit (Xitang Biology Technology Company, Shanghai, China).

### 3.7. Assessment of Infarction Size

At the end of experiment, 2 mL of 5% Evans Blue was injected slowly to the vena cava and used to determine the left ventricular tissue that was not subjected to regional ischemia. The myocardial ischemic area at risk (IAR) was identified as the region without any blue stain. Then the heart was immediately sliced into seven 2-mm thick sections and these were incubated with 5% 2,3,5-triphenyltetrazolium chloride (TTC) at 37 °C for 30 min. The sections were then transferred into 10% formalin and kept at 4 °C for 48 h. The infarction size was defined as TTC-unstained area and expressed as the percentage of the IAR. With respect to clinical importance, only rats with large infarction (> 30%) were selected for analysis [33].

### 3.8. Determination of Oxidant and Antioxidant Enzyme Activities

MDA, SOD, T-AOC and GR as well as GPx in the serum were analyzed using commercially available kits (Nanjing Jiancheng Biological Technology, Nanjing, China) according to the manufacturer’s instructions. All trials were performed in triplicate.

### 3.9. NADPH Oxidase Activity

We measured the activity of Nox, the major source of ROS production. The cardiac Nox activity, expressed as the NADP/NADPH ratio, was measured with a commercial kit (Genmed Scientific Inc. Arlington, MA, USA) by reading absorbance at 340 nm on a microplate reader. The greater the optical density, the higher the Nox activity was determined to be.

### 3.10. Measurement of Superoxide Generation

Production of superoxide was detected in a serial 10 μm frozen sections from LV tissue by DHE (Beyotime Institute of Biotechnology, Nanjing, China) staining. Tissue sections were incubated with 10 μmol/L DHE at 37 °C for 30 min in a humidified chamber protected from light. Fluorescent images obtained with an inverted fluorescence microscope (TE-2000U, Nikon, Japan) and were analyzed with Image-Pro Plus 5.0.

### 3.11. Western Blotting Analysis

Approximately 80 mg of myocardial tissue sample obtained from the LV free wall (anterior wall) was homogeneized in 1 mL lysis buffer containing 50 mmol/L Tris (pH 7.4), 1% TritonX-100, 1 mM EDTA, 0.5% deoxycholic, 500 mM NaCl, 0.1% sodium dodecyl sulphate, 10 mM MgCl_2_, 2 mM Na_3_VO_4_ and protease inhibitors. The homogenate was centrifuged at 12,000*g* for 5 min at 4 °C and the supernatant collected as protein extracts. Protein concentration was determined by Bradford dye reagent (Bio-Rad, Marnes-la-Coquette, France) using bovine serum albumin as standard. Proteins (50 mg) were separated by 10% sodium dodecyl sulphate-polyacrylamide gel electrophoresis and transferred to polyvinylidene fluoride membranes. The membranes were blocked for 1 h at room temperature in 5% dry milk in Tris-buffered saline containing 0.1% Tween 20 (TBST), and incubated overnight at 4 °C with one of the following primary antibodies: rabbit anti-Nox2 (1:2000 dilution, Abcam, Cambridge, UK), rabbit anti-SOD1(1:5000 dilation, Abcam, Cambridge, UK), rabbit anti-SOD2 (1:5000 dilation, Abcam, Cambridge, UK), rabbit anti-nitrotyrosine (1:2000 dilution, Santa Cruz, CA, USA), rabbit anti-p67phox (1:1000 dilution, Cell signaling Technology), rabbit anti-Rac-1 (1:1000 dilution*,* Cell Signaling Technology Inc., Waltham, MA, USA), rabbit anti-PKC-α (1:500 dilution, Bioworld Technology, Inc., St. Louis Park, MN, USA), rabbit anti-phospho-PKC-α (1:500 dilution, Bioworld Technology), rabbit anti-AMPK-1α (1:1000 dilution, Abcam, Cambridge, UK), anti-phospho-AMPK-1α (1:1000 dilation, Abcam, Cambridge, UK) and mouse anti-GAPDH (1:5000, Santa Cruz Biotechnology, Inc., CA, USA). The expression of GAPDH was used as an internal control. After incubation with primary antibodies, the membrane was washed three times with TBST for 10 min each and then incubated for 2 h with appropriate horseradish peroxidase-conjugated secondary antibodies. Chemiluminescence was detected using an ECL-Plus kit (PerkinElmer Life Science, Waltham, MA, USA) and the light signals were detected by X-ray film.

### 3.12. Statistical Analysis

Data were expressed as means ± SEM. Each variable was compared between the different groups using a one-way ANOVA. When an overall difference was found, a Tukey test was performed. A *p*-value of less than 0.05 was set as the criterion for significance in all comparisons.

## 4. Conclusions

**Figure 8:**
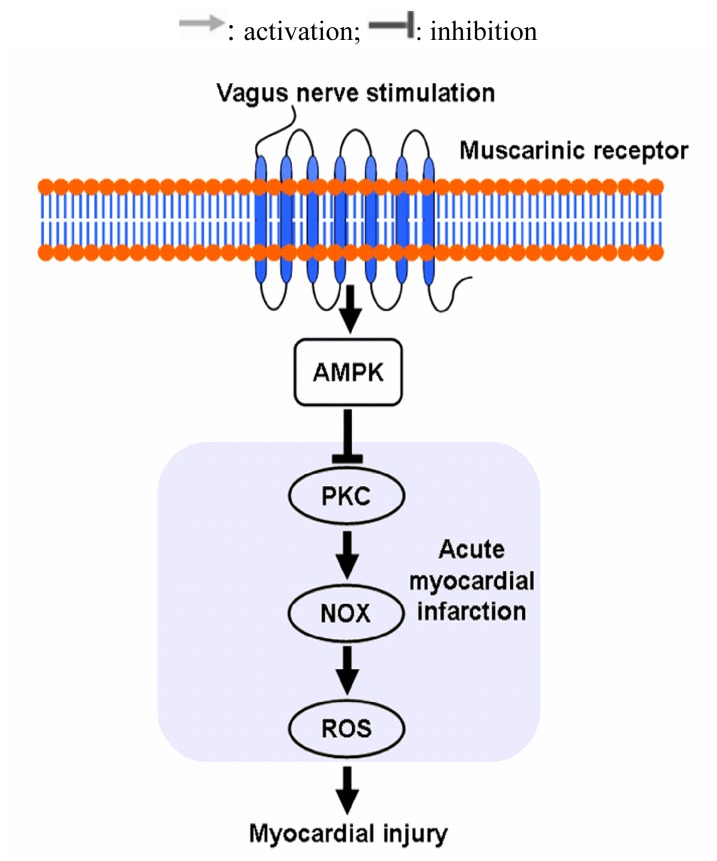
Schematic overview of the protective effects of the VNS on myocardial ischemia-induced ROS production. VNS can inhibit the myocardial ischemia induced-ROS production mainly through the AMPK-PKC-Nox pathway and muscarinic receptor is involved in the cardioprotection role of VNS.

In the present study, we demonstrated how VNS-mediated signaling protects rats with AMI from oxidative stress (Figure 8). We showed that VNS decreased Nox-derived ROS, for the first time, as evidenced by the decreased Nox activity and fluorescence intensity. In addition, this protection was accompanied by the enhanced antioxidant capacity, such as raised plasma T-AOC level and SOD activity as well as its expression. Mechanistically, our results showed for the first time that VNS increased the phosphorylation of AMPK and suppressed the phosphorylation of PKC. Moreover, we also showed that nearly all these cardioprotective roles of VNS were effectively abolished or diminished by atropine, a muscarinic receptor antagonist, implying that muscarinic receptor may participate in the VNS-mediated inhibition of ischemia-induced ROS production. These findings implicate that the parasympathetic nerve system can serve as a promising target for the treatment of ischemic heart diseases.
